# Inter-study variability in CMR measurements of right ventricular volume, mass and ejection fraction in tetralogy of fallot: a prospective observational study

**DOI:** 10.1186/1532-429X-14-S1-P104

**Published:** 2012-02-01

**Authors:** Shannon E Blalock, Puja Banka, Tal Geva, Andrew J Powell, Jing Zhou, Ashwin Prakash

**Affiliations:** 1 Department of Cardiology, Children's Hospital Boston, Boston, MA, USA; 2 Department of Pediatrics, Harvard Medical School, Boston, MA, USA

## Background

Cardiac MRI (CMR) is commonly used for serial monitoring of right ventricular (RV) size and function in patients with repaired tetralogy of Fallot (TOF). However, the inter-study variability of these measurements is not known, which hinders accurate interpretation of serial changes. In this study we prospectively assessed the inter-study variability of CMR measurements of RV size and function in TOF patients.

## Methods

Patients with repaired TOF referred for a clinically indicated CMR examination at our institution were prospectively enrolled. ECG-gated steady-state free precession cine imaging of ventricular long- and short-axis planes was obtained on a 1.5 T scanner. Immediately after the conclusion of the examination, patients returned for a second study performed by a different technologist. Ventricular size and function data from both short-axis image sets were analyzed by a single observer and compared using Bland-Altman analysis with calculation of a repeatability coefficient (2SD of difference between studies), and intra-class correlation coefficient (ICC).

## Results

Between March 2009 and April 2010, 30 patients with repaired TOF (median age 23.5 years, 53% male) were enrolled. Mean RV end-diastolic volume was 158.1±48 ml and mean RV ejection fraction was 57.1±6. Estimates of repeatability coefficient and ICC are summarized in the table. CMR measurements were highly reproducible between studies for quantification of RV end-diastolic volume (repeatability coefficient 24.1 ml, ICC 0.99), ejection fraction (repeatability coefficient 5.8%, ICC 0.94), and mass (repeatability coefficient 9.7 g, ICC 0.93). ICC values were not different between RV and LV for most parameters except mass, for which they were higher for the LV (0.99 vs 0.93, p=0.004). ICC values for all the CMR parameters were not influenced by the presence of a transannular patch.

**Table 1:** Inter-study variability in CMR measurements

	LV		RV		p value comparing ICC between LV/RV
	Repeatability (2SD of difference between studies)	Intra-class correlation coefficient	Repeatability (2SD of difference between studies)	Intra-class correlation coefficient	

End-diastolic volume	17.2 ml	0.98	24.1 ml	0.99	0.8
End-systolic volume	12.3 ml	0.98	17.7 ml	0.98	0.7
Stroke volume	17.0 ml	0.93	21.9 ml	0.95	0.65
Ejection fraction	6.6%	0.84	5.8%	0.94	0.07
Mass	11.6 g	0.99	9.7 g	0.93	0.004

ICC=intra-class correlation coefficient, SD=standard deviation.

## Conclusions

CMR measurements of RV and LV size and function in repaired TOF patients are highly reproducible between studies. These data will aid in interpretation of serial changes and may be useful for sample size calculation in clinical trials.

## Funding

None.

